# Universal Artifacts Affect the Branching of Phylogenetic Trees, Not Universal Scaling Laws

**DOI:** 10.1371/journal.pone.0004611

**Published:** 2009-02-26

**Authors:** Cristian R. Altaba

**Affiliations:** Laboratory of Human Systematics, University of the Balearic Islands, Balearic Islands, Spain; American Museum of Natural History, United States of America

## Abstract

**Background:**

The superficial resemblance of phylogenetic trees to other branching structures allows searching for macroevolutionary patterns. However, such trees are just statistical inferences of particular historical events. Recent meta-analyses report finding regularities in the branching pattern of phylogenetic trees. But is this supported by evidence, or are such regularities just methodological artifacts? If so, is there any signal in a phylogeny?

**Methodology:**

In order to evaluate the impact of polytomies and imbalance on tree shape, the distribution of all binary and polytomic trees of up to 7 taxa was assessed in tree-shape space. The relationship between the proportion of outgroups and the amount of imbalance introduced with them was assessed applying four different tree-building methods to 100 combinations from a set of 10 ingroup and 9 outgroup species, and performing covariance analyses. The relevance of this analysis was explored taking 61 published phylogenies, based on nucleic acid sequences and involving various taxa, taxonomic levels, and tree-building methods.

**Principal Findings:**

All methods of phylogenetic inference are quite sensitive to the artifacts introduced by outgroups. However, published phylogenies appear to be subject to a rather effective, albeit rather intuitive control against such artifacts. The data and methods used to build phylogenetic trees are varied, so any meta-analysis is subject to pitfalls due to their uneven intrinsic merits, which translate into artifacts in tree shape. The binary branching pattern is an imposition of methods, and seldom reflects true relationships in intraspecific analyses, yielding artifactual polytomies in short trees. Above the species level, the departure of real trees from simplistic random models is caused at least by two natural factors –uneven speciation and extinction rates; and artifacts such as choice of taxa included in the analysis, and imbalance introduced by outgroups and basal paraphyletic taxa. This artifactual imbalance accounts for tree shape convergence of large trees.

**Significance:**

There is no evidence for any universal scaling in the tree of life. Instead, there is a need for improved methods of tree analysis that can be used to discriminate the noise due to outgroups from the phylogenetic signal within the taxon of interest, and to evaluate realistic models of evolution, correcting the retrospective perspective and explicitly recognizing extinction as a driving force. Artifacts are pervasive, and can only be overcome through understanding the structure and biological meaning of phylogenetic trees.

Catalan Abstract in Translation S1.

## Introduction

The quest for the Holy Grail inspired great deeds of all sorts, with little use in the end. A current parallel is the search for the Tree of Life, which written in capitals appears to have a Biblical dimension. Indeed, its mythology includes the notion that in such tree one could reach an understanding of life's diversification in the planet. It is thus not too surprising that its search has fired long, acrimonious polemics on the “right” path to truth, eventually looking more like religious wars in the quest of an unattainable dream than scientific arguments in search of the best approximation to reality. Nowadays the field of phylogenetics is healthily moving away from such confrontations, focusing instead in far more fertile avenues of research. However, the temptation of finding in phylogenetic trees the essence of life remains in the eyes of converts.

The superficial resemblance of phylogenetic trees to real branching structures, such as real trees [1] and rivers [2], is at the origin of the quest for general patterns in the shape of phylogenies. Such possibility is indeed intriguing –if this idea had any validity, it should be possible to look for a grand unifying theme in the history of life. Along this line of thought, three recent meta-analyses report to have found regularities in the shape of phylogenetic trees [3]–[5], leading to claims that random models of evolution may explain life's diversification [4], as suggested by early studies [6], or even that there is a “universal scaling in the branching of the Tree of Life”, which would imply that “similar evolutionary forces drive diversification across the broad range of scales” [5]. If this was true, it would indeed be a remarkable finding.

Evolution surely involves linear relationships from parents to offspring, and thus from ancestor to descendant. In order to depict such relationships in print, two-dimensional diagrams are customarily employed, called phylogenetic trees. Thus it seems logical to analyze these trees in order to address macroevolutionary questions [3], [7]–[18]. It is also possible to search for correlates of hierarchical dendritic structures and their properties, such as the relationship between fractal river basins and neutral models of the fish communities inhabiting them [19]. Thus, the geometry of phylogenetic trees deserves indeed a detailed study [14], [20].

However, phylogenetic trees are not real structures. They are almost certainly flawed reconstructions of historical events [14]. Moreover, these trees are just statistical inferences [21]. And most critically, they are calculated without seeking for universal laws and regularities, but instead with the goal of reconstructing particular historical events [22]. It is therefore essential to understand that not all phylogenetic trees have the same value, because they are complex hypotheses. The information content of a such a tree critically depends on at least three points: 1) the quality and quantity of information upon which it is based; 2) the validity of the method used to infer historical relationships; and 3) the fit of the inferred tree to the data. Thus, the worth of a particular phylogenetic tree may range from trivial to substantial, and its accuracy from mere guess to robust hypothesis. A straightforward conclusion is that any meta-analysis of phylogenetic trees performed with no control over their intrinsic merit is subject to severe pitfalls.

In this context, the reported finding of a universal regularity in phylogenetic trees stems from a radical confusion of reality and diagram. Herewith I refute such claims, on the basis that they are based solely on artifact. The idea of universal scaling in phylogenies is completely unwarranted, being instead a consequence of bias in principles and methods. Further developments in the analysis of phylogenetic tree shape should avoid the artifact pitfalls, correcting distortions and reading the paramount signature of biological processes.

## Results

**Figure 1:**
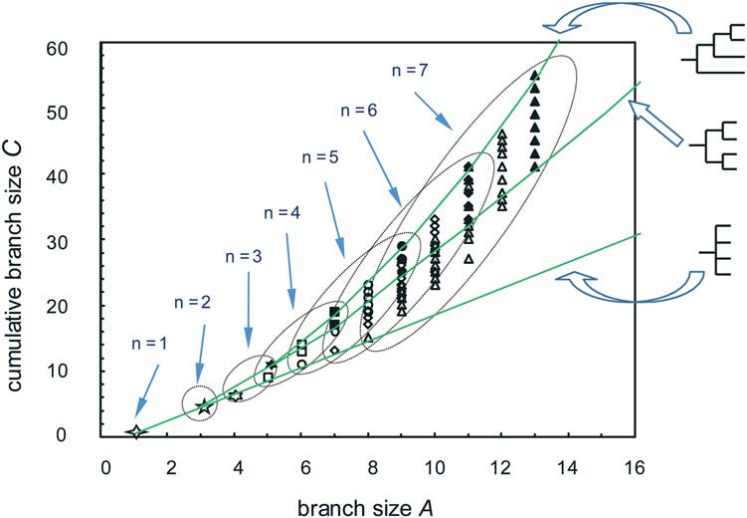
Distribution of rooted, unlabeled trees in tree-shape space, defined by branch size (*A*) and cumulative branch size (*C*). All trees of up to 7 terminal taxa are shown. Solid symbols indicate binary trees, empty symbols stand for non-binary trees. Ellipses encompass all trees with the same number of terminal taxa (n). The lines are the interpolated expectation for three kinds of trees (the 4-taxa examples shown at right): totally symmetrical, random average (middle); pectinate, most imbalanced (top); and totally unresolved, trivial (bottom). The space actually occupied by all trees is limited by the upper and lower bounds. All binary (fully resolved) trees occur at or above the limit imposed by symmetrical trees. Only trees including at least one polytomy (non-binary, or unresolved) occur below this limit.

The distribution of all possible trees in the space defined by *A* and *C* is not random (Fig. 1). All possible trees occur between the bounds imposed by the least and most structured possibilities –fully unresolved and pectinate, respectively. This is an intuitive result, but is relevant because only a small sector of the graph is actually occupied by trees (the remaining regions of the space represent network graphs that are not trees). Trees including polytomies (non-binary, or unresolved) occur throughout this sector. In contrast, all binary trees are bound by a lower limit representing symmetrical trees –i.e., all fully resolved trees lye between two limits: an upper, most structured limit, and a lower one representing average random trees. Thus, any tree located below the symmetrical tree expectation must include at least one polytomy.

**Figure 2:**
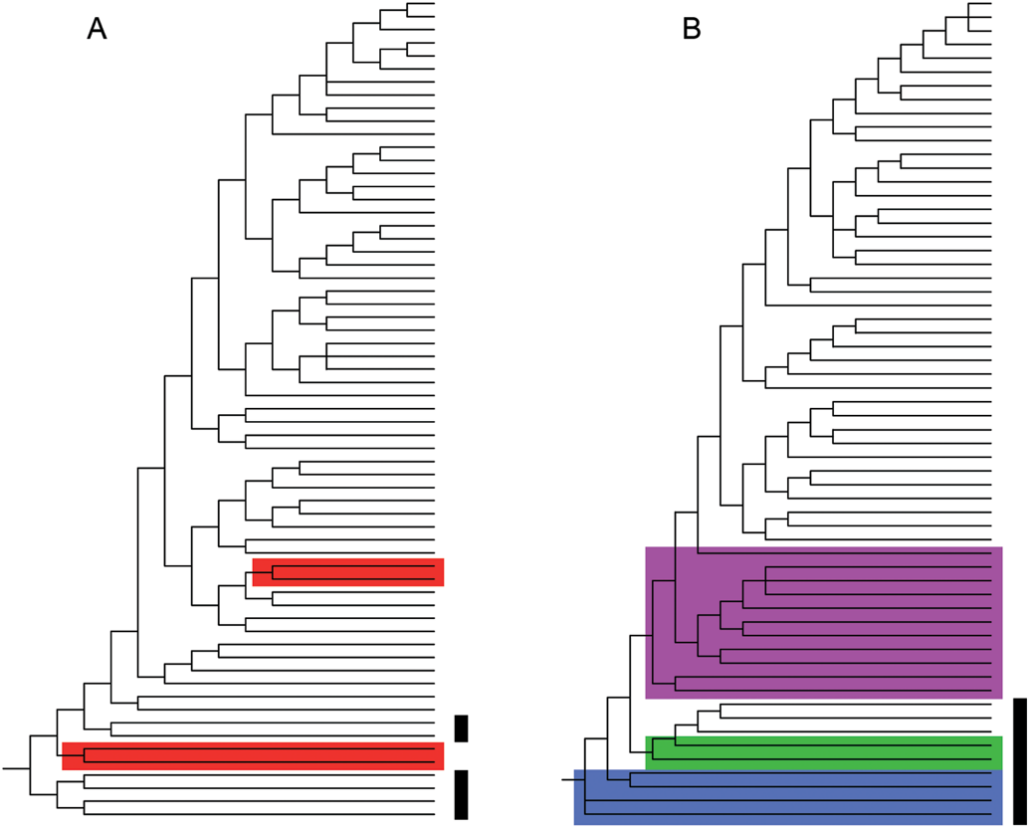
Two analyzed phylogenetic trees, redrawn unlabeled and with uniform internodal distances. A) Fig. 7 from [24]; B) Fig. 1 from [25]. Ingroup taxa are Arachnida and Pectinidae, respectively. Outgroup taxa are marked by thick vertical lines. Basal non-monophyletic taxa are highlighted.

**Figure 3:**
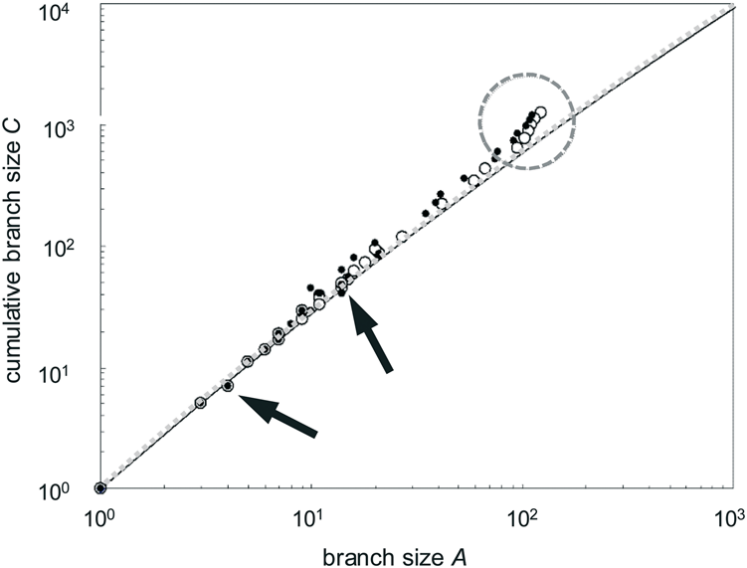
Relationship between branch size (*A*) and cumulative branch size (*C*) throughout two phylogenetic trees (shown in Fig. 2). Each data point represents a node. Notice the logarithmic scale on both axes. Open circles show data for tree A, solid dots stand for tree B. The diagonal line is the interpolated expectation from a random average, totally symmetrical tree. Arrows point at below-expectation values belonging to multifurcations. The dotted circle encloses rapidly diverging values belonging to outgroup and basal paraphyletic taxa.

The relationship between branch size (*A*) and cumulative branch size (*C*) for two analyzed phylogenetic trees (Fig. 2) is shown in Figure 3. At small branch sizes (*A*<10^1^), the data can hardly be distinguished from this expectation, largely due to the narrow band available for small trees. Within a large intermediate section (roughly, 10^1^<*A*<10^2^) the real data are mostly above the symmetrical tree line and span through most of the binary-tree area, indicating that imbalance ranges from null to extreme throughout these real trees. This surely occurs in virtually all real cases, because extinction and unequal rates of speciation occur in the real world and will prevent build-up of perfectly balanced trees. The symmetrical tree (or random model) is thus a baseline useful to measure the degree of imbalance, but cannot be taken as a null model because it represents an unreasonable scenario.

The few data points below the diagonal (indicated by arrows) represent trifurcations in both trees. These non-bifurcating nodes indicate unresolved nodes, the tree-building algorithm being unable to select one of two or more competing hypothesis about binary branching pattern for the three lineages involved. These three-stem nodes are not hypotheses of real multifurcation, being instead purely artifactual.

Near the basal stem of the real trees (roughly, *A*≥10^2^) the values of *C* conspicuously take off, showing that initial branching is most unbalanced in both trees. These deviating, extreme values represent outgroup and non-monophyletic basal taxa. Outgroups are non-arachnid chelicerates (Pycnogonida and Xiphosura) in tree A, and non-pectinids (Limidae, Propeamusiidae and Spondylidae) in tree B. Basal taxa that turn out to be non-monophyletic are the polyphyletic Acari in tree A (highlighted in pink), and the paraphyletic Limidae (blue), Propeamusiidae (green) and Aequipectinini (purple) in tree B. Given that the outgroups were chosen from distantly related taxa, and that poorly defined basal taxa are a heritage of pre-cladistic taxonomy, the deviating values near the root of both phylogenetic trees are just a consequence of method, and are thus purely artifactual.

The resolution provided by the combined use of *A* and *C* is not optimal. The value of *C* is sensitive to the level at which imbalance and polytomies occur. Also, different trees often have the same pair of values. Moreover, both analyzed real trees yield similar scatter plots, in spite of being quite different.

**Figure 4:**
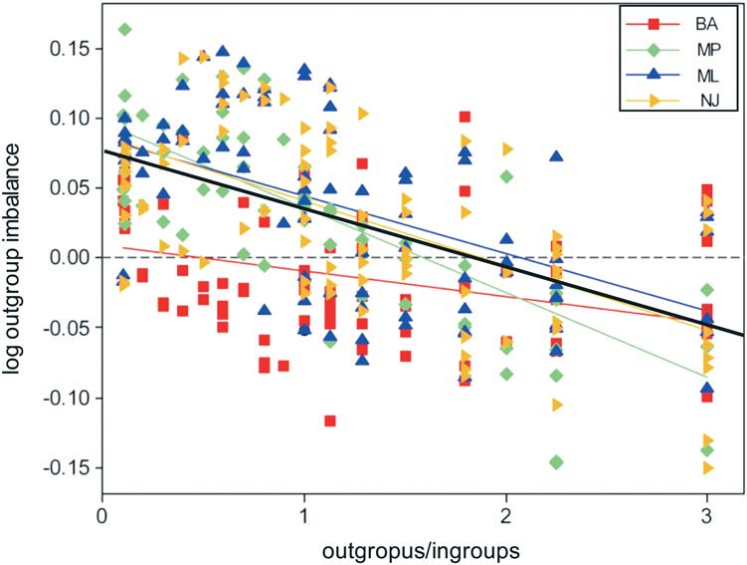
Values of log outgroup imbalance plotted against the relative proportion of outgroups in the dataset of trees obtained applying four tree-building methods to 100 combinations of a set of outgroup and ingroup taxa. Linear regressions are shown for each tree-building method, and for the whole set of 400 trees (thick black line). BA = Bayesian, ML = maximum likelihood, MP = maximum parsimony, NJ = BIONJ distance method.

The 100 combinations of ingroup and outgroup taxa analyzed with four different tree-building methods yielded a non-random relationship between outgroups and the imbalance introduced by these (Fig. 4). The regression of tree imbalance (as measured by log ingroup imbalance) on the proportion of outgroups is highly significant for all four methods, as well as for the whole set of trees (Table 1). However, the regression coefficient ranges from low to moderate, given the wide dispersion of data points. Likewise, the regression slope also varies widely among subsets. The lowest values of *r^2^* and slope are provided by the Bayesian trees, a reflection of their sensitivity to outgroup selection and their tendency to have high node support. At the opposite end, maximum parsimony yields the highest scores for *r^2^* and slope, showing the comparative robustness of this method against variations in the outgroups chosen –parsimony uses outgroups basically to determine character-state polarity. Maximum likelihood and distance methods stand at mid range, probably due to the more algorithm-dependent ways in which they work. Taking all 400 trees together also yields intermediate values, as a result of averaging over the four methods. Pairwise covariance analyses among the four methods show that maximum likelihood and distance regressions are not significantly different, while maximum parsimony and Bayesian are distinct (Table 2).

**Table 1:** Regression analyses for different tree-building methods applied to 100 combinations of a set of outgroup and ingroup taxa.

Data set	equation	*r^2^*	*p*-value
ALL	y = −0.0417x+0.0693	0.356	<0.001
BA	y = −0.0185x+0.0086	0.143	<0.001
ML	y = −0.0413x+0.0854	0.345	<0.001
MP	y = −0.0607x+0.0961	0.672	<0.001
NJ	y = −0.0463x+0.0870	0.417	<0.001

Data sets are all trees (ALL), and trees obained with Bayesian (BA), maximum likelihood (ML), maximum parsimony (MP), and distance (NJ) methods. Variables are the proportion of outgroup taxa (x) and log outgroup imbalance (y). Regression lines are plotted in Fig. 4.

**Table 2:** Pairwise covariance analyses among the different tree-building methods shown in Table 1.

Comparison	x		method		x*method	
	*F*	*p*-value	*F*	*p*-value	*F*	*p*-value
BA vs ML	12.62	<0.001	40.13	<0.001	9.71	<0.005
BA vs ML	17.39	<0.001	71.66	<0.001	45.47	<0.001
BA vs NJ	13.23	<0.001	48.83	<0.001	15.11	<0.001
ML vs MP	66.55	<0.001	0.81	>0.05	7.26	<0.005
ML vs NJ	53.68	<0.001	0.01	>0.05	0.39	>0.05
MP vs NJ	150.46	<0.001	0.61	>0.05	4.19	<0.05

**Figure 5:**
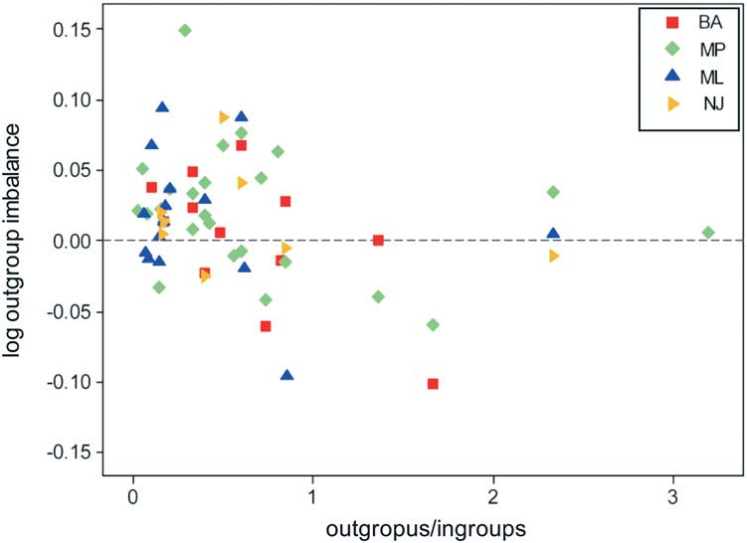
Values of log outgroup imbalance plotted against the relative proportion of outgroups in the dataset of 61 published phylogenetic trees. Data points labeled as in Fig. 4.

The imbalance attributable to outgroups in published phylogenetic trees shows a wide dispersion (Fig. 5). Linear regressions are not significant for maximum parsimony, maximum likelihood and distance-based trees, due to the extreme dispersion of data points. For Bayesian trees, a moderate relationship exists (y = −0.0634x+0.0437, *r^2^* = 0.396, *P*<0.05), but this is probably a spurious result stemming from two artifacts –this method's sensitivity even when few outgroups are included, and the lack in this subset of trees with a high proportion of outgroups. Taking the whole set of published trees, a weak linear regression was found (y = −0.0186x+0.0259, *r^2^* = 0.077, *P*<0.05). However, all values of log outgroup imbalance are normally distributed (mean = 0.0148, s.d. = 0.0436, *AD* = 0.543, *P* = 0.157), suggesting the existence of a constraining factor that keeps real trees close to a situation of null impact of outgroups on tree balance. Although most analyses have few outgroups and these appear to have a low, mostly positive impact on tree balance, values are mostly negative roughly between outgroup proportions around 1 and 2, and above 2 the few data points are close to zero. This suggests a non-linear relationship. Indeed, a quadratic regression (y = 0.0144x^2^−0.0554x+0.0366, *r^2^* = 0.115, *P*<0.05) appears to be slightly better for all published trees. This curvilinear regression suggests that the constraining factor is particularly intense when outgroups clearly outnumber ingroup taxa.

## Discussion

The distressing point from the comparison of different methods of phylogenetic inference is that all of them are quite sensitive to the artifacts introduced by outgroups. The differences among trees obtained with different methods are minor, and appear to be largely related just to the idiosincracy of algorithms. The good news, however, is that published phylogenetic trees appear to be under a remarkable, unexpected constraint. The constraining factor is most likely the fact that practicing taxonomists appear to be generally (and rather intuitively) aware of these artifacts, so they tend to choose carefully the array of outgroup taxa. A corollary of this is that there is no hope for any brute-force meta-analysis performed without consideration of what phylogenetic trees really mean and how they are obtained. A second consequence of this finding is that there is a wide open field for designing formal ways to discriminate the noise due to outgroups from the phylogenetic signal within the taxon of interest. The methods presented here and the following discussion may provide some guide.

Not all phylogenetic trees are equally valid –in fact, there are huge differences in their robustness or support. This variable extent and reliability of phylogenetic hypotheses translates into artifacts in tree shape. For example, poor quality data introduce noise that results in increased imbalance [26]–[28]. Likewise, tree size does have an impact, because real large trees tend to approach a predictable, moderate level of imbalance [4]. These problems can be circumvented in part because tree shape and fit to the data appear to be unrelated [29], and there is at least one measure of imbalance that is independent of tree size [3]. Without being aware of these problems and how to treat them, one may gather a bewildering array of grossly dissimilar trees. Thus, having no control over what different trees mean surely will reduce any possibility of finding common rules.

The three meta-analyses [3]–[5] were based on TreeBASE (http://www.treebase.org), a searchable, archival repository of data and scientific references [30], which can be explored by statistical packages designed to perform large-scale analyses of tree shape [15]. Only binary trees were included in [3], while polytomies were resolved under a random model in [4]. In order to ensure “testing the universality of the results derived across scales”, thousands of cladograms and a few dozen “intraspecific phylogenies” were compiled in [5]. This sampling was totally uncritical, aimed at amassing a bulk of different trees. Moreover, it was partially manual, although simply taking numerous trees with no selection criterion from the literature or from a repository database should yield virtually identical results. Basically, the problem is that it is unclear whether adding numerous hypotheses with an unknown degree of uncertainty may yield a credible global answer.

Resolving phylogenetic trees into perfectly dichotomous branching patterns is a general goal in phylogenetics [31]. However, as any approach that imposes structure on the data, bifurcations are an imposition of method, not necessarily a reality [32]–[35]. All tree-building methods will force a binary tree on the data, but it has seldom been tested at what point of the analysis the conclusions might stretch beyond the assumptions, and thus at what level of detail it would be warranted to stop [21]. One such limitation involves short interior branches (i.e., fast evolutionary radiations), which may be even more prone to error in reality than predicted by theoretical studies [36]. Actually, it may not be really necessary to resolve a multifurcation “bush” (i.e., non-binary splits, or polytomies) in rapidly branching parts of a tree, because the temporal information encoded in that unresolved topology may be more relevant than the detailed sequence of bifurcations [31]. Another overstretching of methods occurs because above species level multifurcations that surely exist in evolution will always tend to be split. A justification may be that it is easier to work on a strictly binary set of nodes, although it is already possible to deal with polytomies in trees [11]. Ideally, the assumptions of systematists should be in agreement with those underlying tree-building algorithms [37]. However, even if there is a real dichotomous structure in the data, unresolved nodes will often occur mostly at or near the terminal branches, because the data analyzed are usually gathered with the goal of resolving mostly the intermediate taxonomic levels considered, and thus may not allow discriminating among very similar terminal taxa. Thus, the best resolution is generally in the middle of published trees. One must bear in mind that awfully unresolved trees are seldom published. Also, it is in the central area that the researcher's interest was in the first place. This explains departures from expected values in the left part of Fig. 3. It is also a good reason to prefer analyses in the tree space defined by *A* and *C*, given that it includes polytomous trees.

The artifactual nature of binary trees is most relevant at or below the species level. Species may be incompletely isolated due to recent or incomplete speciation, the pattern of speciation may not be a simple cladogenetic event but may be instead paraphyletic, hybridization may cause reticulate evolution, and sorting of ancestral polymorphisms may render gene trees incongruent with species trees [17], [38]–[40]. Toward the contemporary tips of a phylogenetic tree, resolution is subject to the delimitation of species, a complex and often arbitrary issue that is not part of the phylogenetic inference process; eventually, recognizing the distinctiveness of individual taxa becomes problematic, because recent and incipient speciation may be difficult to identify [17], [41]. Even more problematic is portraying intraspecific variation as a branching tree. Within a species there is gene flow, so gene trees will most rarely be amenable to be translated directly into a history of population subdivision. It would be more meaningful to ask in the first place if there is an inherent hierarchical structure in data [34]. Actually, the clustering of subpopulations and the comparison of trees for different genes are by no means simple tasks, and dichotomous branching ordinations are just a small part of the methods available [42]. However, being aware of their meaning, they can be powerful tools in combination with other approaches to deal with intraspecific data [13], [43]. It is obvious that trees of intraspecific variation are actually simplified sketches, and thus have a radically different nature than interspecific trees. Thus, the mixing of intraspecific and interspecific trees in [5] has no justification, and their claims of uniform branching pattern above and below the species level are simply an artifact of applying similar binary-tree-building methods to different biological questions. At any rate, the high prevalence of multifurcations that exists among intraspecific trees reflects the inadequacy of tree-building methods for reticulate data, and their finding of lower-than expected values of *C* at short branch lengths is solely an artifact.

The selection of trees is also a source of noise. In fact, different tree-building methods produce significantly different arrays of trees [3], [44]. This precaution was not taken into account by [4], [5], who mixed trees obtained from various kinds of tree-building algorithms –some distance-based (neighbor-joining), some based on parsimony, and still others on maximum likelihood. The differences between these methods can be shown to be rather of “degrees of freedom” [21], [45], yet they are based on different assumptions and often yield different outcomes for the same data matrix (as shown in Fig. 4). Moreover, real-world deviations from theoretical simple models of evolution may easily produce artifactual phylogenetic reconstructions under the commonly used models of sequence evolution, and it is still unclear how to capture the historical signal with a minimum of parameters to be estimated from the data [46]–[48]. Also, trees may differ if calculated with a naïve one-step process, or are derived from an approach that seeks to compare trees and find an average final model [20], [21] –even in simple 3-taxon cases, the outcome may differ strikingly, with substantial evolutionary implications [49]. Thus it remains unclear why trees obtained with different methods from a variety of taxa should be mixed up with no control.

The value of a null model lies not in its mathematical elegance, but in its relevance to the question posed. On average, a totally balanced tree is also expected from Yule's equal-rates Markov model [3], [50], [51], but this kind of tree would be most unusual for any large set of real taxa. In the case of phylogenetic trees, null models based on random, increasing, balanced diversification [5], [6] were only a reasonable early start. More elaborate stochastic models exhibit an enhanced approach to real trees [3], [4], but it is unclear whether there is any reason to prefer any such model beyond a rough fit to the data and the rejection of the overly simplistic Yule model. Clearly, more realistic models are needed that place randomness right where relevant variables impact the model's behavior [16], [17], [52]–[54]. From this viewpoint, it should come as no surprise the finding in all three meta-analyses [3]–[5] that the average imbalance of phylogenetic trees inferred from real data falls neatly in between extreme possibilities (i.e., the symmetric and pectinate trees in [5]; the random and uniform models in [3]; and random and pectinate trees in [4]).

The departure of real trees from random models can be caused at least by two major natural factors, and two artifacts. The first natural factor is simply that extinction does occur, so not all lineages can continue to divide at the specified rate. As lineages go extinct along a tree, its imbalance will almost inevitably increase. This is a consequence of extinction being the outcome of complex dynamics, so it is not reasonable to expect that it should remain stable across the tree. The second natural deviating factor is that diversification rates will surely vary across the different branches of the tree over time, because it is a complex function of a plethora of intrinsic and environmental factors operating on living organisms. Several methods have been devised to estimate absolute rates of speciation and extinction, showing that large variation in those parameters is the rule [55]–[63]. Indeed, balanced random processes are too slow to account for most patterns of observed diversity, yet diversification is subject to complex environmental constraints [17], [53]. A reflection of such complexity is likely to result in autocorrelation of diversification rate along lineages [8]. Thus, real phylogenies should be expected to range throughout all possible topologies, with no reasonable way of *a priori* delimiting tree space.

Aside from real-world issues, the two major artifacts that increase imbalance are related to the taxa included in the analysis. On one hand, all known taxa from a given group are rarely included, so some choice has to be made. Often this may be imposed by the availability of samples. However, it may be difficult to know whether species have been removed from the analysis deliberately and selectively [26]. And including selected species from high-rank taxa may cause problems of two sorts. Actually, real trees are quite imbalanced, and more so if the taxa are above the species level [39]. In addition, such large branches will inevitably result in underestimation of real change, and thus of long branch lengths. This is the pervasive node-density artifact, whose impact on tree shape is still unclear [64]. At any rate, non-random taxon sampling will cause errors in estimates of speciation and extinction rates, more so than just incomplete taxon sampling [65], [66]. Indeed, the inclusion of evolutionarily isolated species may affect synthetic measures of phylogenetic trees [67].

On the other hand, outgroups (used to place the root of the tree) are a definite source of imbalance. At the highest taxonomic levels considered, *C* has higher-than-expected values, indicating that long branches tend to be more pectinate. But this is due to the inclusion of selected taxa from progressively more distantly related lineages. This is routinely done in order to provide various outgroups. This is justified because, based on sampling theory, the more dense the sampling of outgroup taxa, the more stable the internal topology will be and the stronger the test for the monophyly of the ingroup [68], [69]. Being clear that outgroup taxa significantly contribute to an excess of imbalance [3], [21], [70], there is a motive for removing outgroups from tree analysis [3], [4]. Unfortunately, the outgroup taxa are often not displayed in the published trees, and it is frequent that more outgroups are included than those explicitly identified as such. Actually, outgroups often involve more than just the first low-diversity branch, or the usual basal one or two single-species branches. In some instances, such as in tree A (Fig. 2), a priori outgroups turn out not to be the branches closest to the root, making any automated identification and deletion of outgroups highly suspect. This problem is exacerbated if the basal taxa turn out to be paraphyletic [39], because they will appear as pectinate long branches. The two trees analysed in detail (Fig. 3) show several basal branches that belong to outgroups that are revealed to be paraphyletic. Actually, higher taxa that have traditionally been considered as basal to other higher-order taxa often turn out to be paraphyletic when subject to cladistic evaluation –the Acari, Limidae, Propeamussiidae and Aequipectinini are likely candidates to join the club of outfashioned, unnatural groups such as the Protobranchia, Reptilia, and Pongidae. Without a proper identification of outgroup taxa, coupled to a taxonomic assessment of any basal paraphyletic taxa, it is very hard to control for the pervasive artifact of imbalance increasing at the highest taxonomic levels of published trees. Therefore, the reported findings of imbalance increasing at large tree sizes stems from this control being insufficient in [4] and just missing in [5], and thus appears to be totally caused by the outgroup and basal paraphyly artifacts.

Various tree-shape statistics have been divised, whose merits vary widely. Most of these methods extract a single summary index from the distribution of nodes, so it's not too surprising that the majority of such measures of tree shape are sensitive to the level, or depth in the phylogeny at which imbalance is concentrated [3], [71] and to the presence of polytomies [36]. As summarized in Fig. 1, *C* suffers from these same shortcomings. Focusing instead on the dispersion of node traits in a bidimensional plot aims at capturing more of the tree's features [72], although interpretation of such analysis is also difficult [3], [10]. Likewise, estimates of the alpha model fail to adjust extreme tree shapes and often yield a zero value [3], thus being also hard to interpret. As shown above, the relationship between *A* and *C* can be used to locate and explain imbalance in the different regions of a given tree, even if there are polytomies. The drawbacks of this method are that it does not have optimal resolution because different trees yield identical values, and all trees are constrained within a small sector of geometric space, so even quite distinct trees will yield similar plots. Nevertheless, it is clear that the two phylogenies in Fig. 2 have quite different shape, yet are translated into overall similar plots in Fig. 3. It is also relevant to notice that these two parameters can be used to design meaningful measures (such as log outgroup imbalance) of the impact of outgroups (and possibly other artifacts) in tree space. Thus, the uniform relationship among branch size *A* and cumulative branch size *C* is due to a narrow design of methods, not a quality of results.

A third avenue is to compare trees strictly in terms of what they are –high-dimensional parameters amenable to geometrical depictions in ultrametric space [73]. Actually, ultrametrics have been successfully applied to a variety of questions where data have a hierarchical structure [34], [74], [75]. This perspective allows the exploration of geometric space [14], [20], [76], without relying on simulations, and leading to the application of statistical methods [21]. It is thus possible to develop a measure of resolution for different tree-shape statistics, and thus select those statistics that have similar values only for similar trees [14]. The analysis shown in Fig. 1 is a step in this direction, pointing at further developments in generalized tree shape distribution.

However, there is a critical caveat to any analysis of the shape of phylogenetic trees. Our perspective being inevitably from the present, extant diversity always appears to come out of a burst from a distant single stem [17]. Virtually all real trees will have a rather “conical” shape, due to the fact that the recent splits considered are many more than old surviving lineages. Including extinct taxa should help in correcting this retrospective illusion, but the incompleteness of the fossil record will always play against such correction. But this leads to a second obstacle, which is related but more difficult to tackle –what exactly are fossil taxa that are basal to later diversification. In an orthodox cladistic framework, such an extinct species will always be treated as the sister group of all later branches, provided the traits of later taxa can be inequivocally identified in their earliest stages. Now, this methodological shortcut may not always provide an accurate description of reality, our placing of those early stems, or “species germinalis”, being strongly dependent on later evolution that is only apparent from our contemporary point of view [77]. Clearly there is a challenge to develop methods for correcting our “convex from the present” view of phylogenetic trees prior to analysis of their actual shape and information content.

In spite of grand declarations, the Darwinian goal of classifying organisms in terms of their relationships of common descent has powered evolutionary research and is at the root of the field of phylogenetics. There is really nothing like universal scaling in phylogenetic trees –and no good reason why it should exist. We are dealing with attempts to understand history [22], thus a phylogenetic tree is only a diagram of a complex irreversible process. In this sense, the linking of TreeBASE to databases providing information on the taxa actually included in each analysis [78] is a valuable addition that should help in assessing the significance and merits of each tree before including it in any meta-analysis. Beyond failures based on unreasonable assumptions and oversimplistic paradigms, the wealth of information encoded in phylogenetic trees is there to be deciphered. However, this will not happen with any uncontrolled meta-analysis, but only through an integration of population genetics, ecology, paleontology, and graph theory. Artifacts pave the way, and they can only be overcome with an understanding of the structure and biological meaning of phylogenetic trees.

Exploring the geometry of unlabeled trees with constant internodal distances represents only an initial approach. It is critical to notice that taking tree topologies alone explicitly disregarding any time scale has the implicit problem of obviating extinction. Actually, time on a phylogeny does matter, at least because individual branch lengths actually are estimates of different processes depending on where they are located within the tree. Towards the terminal taxa, individual branch lengths estimate the inverse of the speciation rate, but at the basal regions they rather estimate the inverse of the diversification rate, being the difference between the speciation and extinction rates [79]–[81]. It may even be possible to distinguish decreasing speciation from increasing extinction in early evolutionary radiations [63]. This is relevant to methods such as the lineage-through-time approach [82], [83], which ignores extinct lineages and is thus sensitive to the effects of poor sampling of taxonomic diversity, as well as to its intrinsic inability to distinguish reduced extinction and enhanced speciation [17]. Although the variability of branch lengths in real trees can be used to test hypothesis about evolutionary rates [65], [84], precise estimation of these rates requires large phylogenetic trees [85], and it is still unclear how to assess in general the impact of disappearing lineages on the shape of phylogenetic trees. Although it is episthemologically impossible to read directly the empty space left by vanished taxa, the contribution of missing branches to the observed patterns remains as a signature to be deciphered. Eventually, it is the biological phenomenon of extinction that imposes an ultrametric structure on phylogenetic trees, because the unavoidable disappearance of interfertile individuals and intermediate taxa throughout life's history sets apart the surviving lineages and promotes the growth of biodiversity.

## Materials and Methods

All rooted, unlabeled trees consisting of up to 7 terminal branches (unnamed taxa) were enumerated, separating binary (fully resolved) trees from those having at least one polytomy (i.e., having one unresolved node). Among the variety of indices devised to sumarize tree shape, the values of branch size (the number of subtaxa from a given node, *A*) and cumulative branch size (the sum of the sizes of all branches from a given node, *C*), the two variables measured in [5], were manually calculated for each tree. In order to explore the distribution of all trees in an *A* vs. *C* plot (Fig. 1), these values were calculated also for three series of trees: perfectly symmetrical trees, which are expected on average from a purely random branching process; pectinate trees, which are most imbalanced; and totally unresolved trees, being the trivial bottom-line with one single node. Each series was drawn as a line; this is a continuous interpolation that allows drawing a simple limit in this tree space [23].

Two data-rich phylogenetic trees were selected from recent literature: Fig. 7 in [24], and Fig. 1 in [25]. They belong to different phyla (Arthropoda and Mollusca) and different environments (terrestrial and marine, respectively). Both include only distinct species (i.e., there are only undisputed individual terminal branches), are relatively large (≥60 terminal taxa), include several outgroups and non-monophyletic basal taxa, are the product of excellent scholarship on DNA sequences, and are considered by their authors as working hypotheses likely to change with the inclusion of further evidence. They are shown in Figure 2, redrawn in order to depict only their topology. Tree A is more balanced near the terminal taxa, while tree B is more balanced near the root. The values of *A* and *C* were calculated for all subtrees in both trees. A log-log plot of *A* vs. *C* was drawn in order to show deviations from the symmetrical tree expectation (Fig. 3).

In order to explore the variation in the relationship between *A* and *C* in relation to the proportion of outgroups included in phylogenetic analyses, different tree-building methods were applied to various combinations of a given set of ingroups and outgroup taxa. The aminoacid sequences included in this analysis belong to the AAA (ATPases Associated with a wide variety of cellular Activities) protein (either replication factor C small subunit, or DNA polymerase III gamma subunit), introduced as example in the Phylogeny.fr [86] data window (viruses excluded): 10 eukaryots considered as ingroup taxa, and 9 prokaryots (4 Eubacteria and 5 Archaea) taken as outgroups. The species considered are (followed by accession number in the Entrez database: http://www.ncbi.nlm.nih.gov/sites/entrez?dbprotein): *Plasmodium chambaudi* (XP_745209), *Trypanosoma brucei* (XP_829019), *Dictyostelium discoideum* (XP_629875), *Schizosaccaromyces pombe* (NP_593121), *Ustilago maydis* (XP_756876), *Arabidopsis thaliana* (NP_176504), *Caenorhabditis elegans* (NP_500069), *Anopheles gambiae* (XM_308395.4), *Strongylocentrotus purpuratus* (XP_790650), *Homo sapiens* (NP_002905), *Aquifex aeolicus* (NP_214275), *Polaribacter irgensii* (ZP_01118896), *Ehrlichia ruminantium* (YP_196867), *Neisseria meningitidis* (NP_284372), *Methanosarcina acetivorans* (NP_615630), *Haloarcula marismortui* (YP_137064), *Halobacterium* species NRC-1 (NP_280914), *Methanosphaera stadtmanae* (YP_447457), and *Methanospirillum hungatei* (YP_502463). A total of 100 combinations of ingroup and outgroup taxa were selected, spanning throughout all possible values of the ingroup/outgroup ratio. For each combination of taxa, an independent analysis was performed using the Phylogeny.fr platform (http://www.phylogeny.fr). Sequences were aligned with MUSCLE [87], and phylogenetic trees were estimated through four different methods: Bayesian approach using MrBayes (ver. 3.1.2) [88] with GTR option for substitution types, invariable and gamma rate variation across sites; maximum likelihood using PhyML (ver. 3.0 aLRT) [89], [90]; maximum parsimony as implemented in TNT (ver. 1.1) with sectorial search and tree fusing [91], [92]; and distance analysis using BIONJ [93]. The Bayesian analyses included a Monte Carlo Markov Chain with 10,000 generations, sampling a tree every 10 generations, and discarding the first 250 trees sampled as burn-in. The other three methods involved 100 bootstrap replicates, yielding strict consensus trees. Nodes with support values below 50% were collapsed. The root was placed between the Archaea and the Eubacteria (or in rare cases the group formed by these and one archaeon). Values of *A* and *C* were calculated manually for each of the 400 resulting trees and their ingroup set. The difference in the *C/A* ratio (taking logarithmic values) between the whole tree and after deleting the outgroups is called log outgroup imbalance, and is a measure of the change in relative position within the tree space defined by these two variables (shown in Fig. 2). Thus, a positive value means a steeper position of the whole tree relative to the ingroup set for the position in that tree space, due to a positive contribution of the outgroups to tree imbalance. A negative value means a drop in relative position when outgroups are considered, meaning that outgroups actually decrease tree imbalance. The values of log outgroup imbalance were plotted against the relative proportion of outgroups in the dataset (Fig. 4). Linear regressions were calculated for the whole set of 400 trees, and separately for those obtained by each tree-building method. These regressions were compared pairwise through analyses of covariance.

In order to test whether the relationship found occurs in other datasets, a total of 61 published phylogenies (including the two already analyzed) were selected (Table 3) [94]–[125]. This is an explicitly ecclectic selection of studies, based on the variety of my interests and readings. It is no more arbitrary than a random download from a database of phylogenetic trees, and no less rigorous than a well-posed query to it –actually it is more reliable because trees were selected only after scrutiny of the actual papers where they have been published. The species involved span throughout a wide variety of eukaryots, and the supraspecific ingroup taxa range from a single genus to a whole class. The data are only nucleic acid sequences, and the period of publication is the last 11 years. Most papers provided one tree, although in several instances the same dataset was analyzed with different methods, and different methods are sometimes applied to different datasets. Thus, every tree sampled is taken as independent. Only species-level taxa were considered; thus whenever populations belonging to the same (sub)species represented different branches these were united. Nodes with support values below 50% were collapsed. In all cases, the outgroups were those actually included in the analysis –this is often clear in the illustrated phylogenetic trees, but in a few papers it is only evident in the text. Values of *A* and *C* were calculated manually for each of the 400 resulting trees and their ingroup set, and log outgroup imbalance was plotted against the relative proportion of outgroups in the dataset (Fig. 5). The fit of all these log outgroup imbalance values to a normal distribution was tested with the Anderson-Darling goodness-of-fit statistic. Linear and quadratic regressions were calculated for the whole set of published trees, as well as for subsets of trees obtained with different methods.

**Table 3:** Published phylogenetic trees analyzed. Trees are ordered by method of inference (BA = Bayesian, ML = maximum likelihood, MP = maximum parsimony, NJ = distance), proportion of outgroups relative to ingroup taxa (out/in), and log outgroup imbalance (LOI). Values of *A* and *C* are given for the complete trees and for ingroup taxa only.

method	in	out	out/in	*A* all	*C* all	*A* in	*C* in	LOI	taxa	
BA	19	2	0.1053	39	299	35	221	1.3524	terrestrial pulmonates	[94]
BA	6	1	0.1667	13	55	11	41	0.5035	passerine birds	[95]
BA	85	28	0.3294	215	2457	161	1255	3.6329	lower neopterous insects	[96]
BA	3	1	0.3333	7	19	5	11	0.5143	terrestrial caenogastropods	[97]
BA	10	4	0.4000	26	115	18	72	0.4231	cichlid teleosts	[98]
BA	35	17	0.4857	100	994	68	543	1.9547	centaurine composites	[99]
BA	5	3	0.6000	13	45	8	19	1.0865	passerine birds	[100]
BA	34	25	0.7353	116	905	67	533	0.1535	carnivore mammals	[101]
BA	34	28	0.8235	122	923	67	417	1.3417	pancrustaceans	[102]
BA	13	11	0.8462	45	310	24	110	2.3056	aquatic pulmonates	[103]
BA	25	34	1.3600	116	905	48	256	2.4684	carnivore mammals	[101]
BA	3	5	1.6667	14	39	5	11	0.5857	plethodontid salamanders	[104]
ML	17	1	0.0588	35	245	33	209	0.6667	mammals	[105]
ML	15	1	0.0667	30	113	29	111	0.0609	terrestrial pulmonates	[106]
ML	12	1	0.0833	24	97	23	95	0.0888	aquatic caenogastropods	[107]
ML	40	4	0.1000	82	681	74	438	2.3860	mammals	[108]
ML	14	2	0.1429	27	93	24	83	0.0139	procellariiform birds	[109]
ML	54	8	0.1481	121	920	106	755	0.4807	pancrustaceans	[102]
ML	25	4	0.1600	51	425	43	229	3.0078	terrestrial pulmonates	[94]
ML	6	1	0.1667	13	55	11	41	0.5035	passerine birds	[95]
ML	50	9	0.1800	112	1200	95	838	1.8932	pectinid bivalves	[25]
ML	15	3	0.2000	28	105	23	71	0.6630	nemerteans	[110]
ML	10	4	0.4000	24	86	16	45	0.7708	cichlid teleosts	[98]
ML	5	3	0.6000	15	67	9	25	1.6889	passerine birds	[100]
ML	26	16	0.6154	72	485	44	256	0.9179	rodent mammals	[111]
ML	7	6	0.8571	21	83	13	53	0.1245	perameloid marsupials	[112]
ML	3	7	2.3333	16	63	5	11	1.7375	insectivore mammals	[113]
MP	44	1	0.0227	89	735	87	645	0.8446	decapod crustaceans	[114]
MP	38	2	0.0526	78	337	75	257	0.8938	vetigastropods	[115]
MP	25	2	0.0800	48	324	45	274	0.6611	anguid lizards	[116]
MP	14	2	0.1429	30	163	27	155	0.3074	procellariiform seabirds	[109]
MP	13	2	0.1538	27	133	24	104	0.5926	aquatic caenogastropods	[117]
MP	6	1	0.1667	13	55	11	41	0.5035	passerine birds	[95]
MP	23	4	0.1739	38	322	30	211	1.4404	conifers	[118]
MP	15	3	0.2000	27	103	22	69	0.6785	nemerteans	[110]
MP	14	4	0.2857	28	168	20	64	2.8000	pond turtles	[118]
MP	85	28	0.3294	214	3057	159	1657	3.8637	lower neopterous insects	[96]
MP	21	7	0.3333	40	184	31	125	0.5677	pond turtles	[119]
MP	10	4	0.4000	23	79	15	39	0.8348	cichlid teleosts	[98]
MP	10	4	0.4000	27	137	19	77	1.0214	passerine birds	[120]
MP	38	16	0.4211	87	569	61	327	1.1796	rodent mammals	[111]
MP	14	7	0.5000	41	297	27	125	2.6143	amphibians	[121]
MP	18	10	0.5556	50	265	31	139	0.8161	juglandaceans	[122]
MP	10	6	0.6000	31	145	19	73	0.8353	anseriform birds	[123]
MP	5	3	0.6000	13	46	8	19	1.1635	passerine birds	[100]
MP	7	5	0.7143	22	89	12	33	1.2955	unionoid bivalves	[124]
MP	34	25	0.7353	114	888	66	484	0.4561	carnivore mammals	[101]
MP	35	28	0.8000	123	1250	95	640	0.0629	arachnids	[24]
MP	13	11	0.8462	45	275	24	114	1.3611	freshwater pulmonates	[103]
MP	25	34	1.3600	114	888	47	290	1.6193	carnivore mammals	[101]
MP	3	5	1.6667	12	35	5	11	0.7167	plethodontid salamanders	[104]
MP	3	7	2.3333	18	82	5	11	2.3556	insectivore mammals	[113]
MP	5	16	3.2000	37	187	21	81	1.1969	passerine birds	[120]
NJ	13	2	0.1538	28	143	25	113	0.5871	aquatic caenogastropods	[117]
NJ	37	6	0.1622	79	512	68	405	0.5251	protochordates	[125]
NJ	6	1	0.1667	13	55	11	41	0.5035	passerine birds	[95]
NJ	10	4	0.4000	24	99	16	59	0.4375	cichlid teleosts	[98]
NJ	14	7	0.5000	41	285	27	113	2.7660	amphibians	[121]
NJ	5	3	0.6000	15	59	9	25	1.1556	passerine birds	[100]
NJ	13	11	0.8462	41	195	22	82	1.0288	aquatic pulmonates	[103]
NJ	3	7	2.3333	17	66	5	11	1.6824	insectivore mammals	[113]

## Supporting Information

Translation S1 Abstract translated into Catalan
(0.03 MB DOC)
