# Supplementary material for: Universal Artifacts Affect the Branching of Phylogenetic Trees, Not Universal Scaling Laws
Source: PLoS One. 2009 Feb 26;4(2):e4611. doi: 10.1371/journal.pone.0004611 (PMC2644784; doi:10.1371/journal.pone.0004611)
Supplement: Translation S1 — Abstract translated into Catalan (0.03 MB DOC) [file pone.0004611.s001.doc]

## Artefactes universals afecten la ramificació dels arbres filogenètics, no lleis d’escalatge universal

## Cristian R. Altaba

Laboratori de Sistemàtica Humana, Ed. Ramon Llull, Universitat de les Illes Balears, 07071 Palma de Mallorca, Illes Balears, Espanya. cristianr.altaba@uib.es

## Resum

**Antecedents**

La semblança superficial dels arbres filogenètics a d’altres estructures ramificades permet cercar-hi patrons macroevolutius. Ara bé, aquests arbres són només inferències estadístiques d’esdeveniments històrics particulars. Algunes metaanàlisis recents anuncien haver trobat regularitats en el patró de ramificació dels arbres filogenètics. Però està això recolzat per l’evidència, o bé aquestes regularitats són només artefactes metodològics? Si és així, hi ha cap senyal en una filogènia?

**Metodologia**

Amb l’objectiu d’avaluar l’impacte de les politomies i del desequilibri sobre la forma dels arbres, es determinà la distribució de tots els arbres binaris i politòmics de fins a 7 tàxons en l’espai de formes d’arbre. La relació entre la proporció de grups externs i el grau de desequilibri que s’introdueix amb aquests s’avaluà aplicant quatre mètodes diferents de construcció d’arbres a 100 combinacions d’un conjunt de 10 espècies com a grups interns i 9 com a externs, i realitzant anàlisis de covariança. La rellevància d’aquesta anàlisi s’explorà considerant 61 filogènies publicades, basades en seqüències d’àcids nucleics i amb implicació de diversos tàxons, nivells taxonòmics i mètodes de construcció d’arbres.

**Troballes principals**

Tots els mètodes d’inferència filogenètica són força sensitius als artefactes introduits pels grups externs. Ara bé, les filogènies publicades semblen subjectes a un control bastant efectiu, encara que més aviat intuïtiu, damunt aquests artefactes. Les dades i els mètodes emprats per construir arbres filogenètics són diversos, la qual cosa fa que qualsevol metaanàlisi estigui subjecta als riscs deguts als seus mèrits desiguals, que es tradueixen en artefactes en la forma dels arbres. El patró de ramificació binària és una imposició dels mètodes, i rarament reflecteix les veritables relacions intraespecífiques, donant lloc a politomies artefactuals en arbres curts. Per damunt del nivell d’espècie, la desviació dels arbres reals enfora de models aleatoris simplistes està causada per almenys dos factors naturals (els ritmes heterogenis d’especiació i d’extinció) i artefactes com ara la tria de tàxons inclosos a l’anàlisi, el desequilibri introduït pels grups externs i els tàxons parafilètics basals. Aquest desequilibri artefactual explica la convergència en la forma dels arbres grans.

**Rellevància**

No hi ha evidència de cap escalatge universal en l’arbre de la vida. Calen, en canvi, mètodes millorats per a l’anàlisi d’arbres que es puguin emprar per discriminar el soroll degut als grups externs del senyal filogenètic a l’interior del tàxon d’interès, i per avalaur models realistes d’evolució, tot corregint la perspectiva retrospectiva i reconeixent explícitament l’extinció com una força motriu. Els artefactes són omnipresents, i només es poden superar mitjançant la comprensió de l’estructura i el significat biològic dels arbres filogenètics.
